# Structural features in the Rous sarcoma virus RNA stability element are necessary for sensing the correct termination codon

**DOI:** 10.1186/1742-4690-7-65

**Published:** 2010-08-05

**Authors:** Johanna B Withers, Karen L Beemon

**Affiliations:** 1 Department of Biology, Johns Hopkins University, 3400 N. Charles St., Baltimore, MD 21218, USA

## Abstract

**Background:**

Nonsense-mediated mRNA decay (NMD) is an mRNA quality control mechanism that selectively recognizes and targets for degradation mRNAs containing premature termination codons. Retroviral full-length RNA is presented to the host translation machinery with characteristics rarely observed among host cell mRNAs: a long 3' UTR, retained introns, and multiple open reading frames. As a result, the viral RNA is predicted to be recognized by the host NMD machinery and degraded. In the case of the Rous sarcoma virus (RSV), we identified a stability element (RSE), which resides immediately downstream of the *gag *termination codon and facilitates NMD evasion.

**Results:**

We defined key RNA features of the RSE through directed mutagenesis of the virus. These data suggest that the minimal RSE is 155 nucleotides (nts) and functions independently of the nucleotide sequence of the stop codon or the first nucleotide following the stop codon. Further data suggested that the 3'UTRs of the RSV *pol *and *src *may also function as stability elements.

**Conclusions:**

We propose that these stability elements in RSV may be acting as NMD insulators to mask the preceding stop codon from the NMD machinery.

## Background

Nonsense-mediated mRNA decay (NMD) selectively recognizes and targets for degradation mRNAs containing premature termination codons. This mRNA quality control mechanism prevents potentially deleterious dominant negative effects of truncated proteins that accumulate if aberrant mRNAs are not degraded [1-4]. In mammalian cells, NMD proteins can efficiently identify a termination codon as premature if the stop codon resides at least 50 nucleotides upstream of the terminal exon-exon junction [5,6].

When introns are removed during splicing, a multi-protein complex called the exon junction complex (EJC) is deposited on the mRNA 20-24 nucleotides upstream of the exon-exon junction [7]. When a translating ribosome encounters a termination codon, it pauses; and the eukaryotic release factors, eRF1 and eRF3, as well as the NMD factors Upf1 and Smg1, are recruited [8]. If the termination codon is premature, Upf1 will interact with the downstream EJC via two additional NMD factors, Upf2 and Upf3b. This forms a decay-inducing complex that signals a premature termination event [8]. The mRNA is then rapidly targeted for degradation in the cytoplasm so that it is no longer translated. In most mRNA transcripts, the natural termination codon resides in the final exon of a spliced transcript, preventing the occurrence of a downstream EJC [9].

NMD poses a unique risk to the genome and mRNAs of retroviruses. Although retroviruses encode some enzymatic activities, they rely on the host cell's reservoir of proteins to produce progeny virions. As a result of this dependence on host cell machinery, retroviruses must overcome mRNA quality control measures to ensure their genome is translated in an efficient and timely manner. The genomes of simple retroviruses, such as the Rous sarcoma virus (RSV), possess cis-acting RNA elements that play an essential role in facilitating successful genomic expression [10-13].

During the RSV life cycle, expression of the integrated proviral DNA generates three viral mRNAs that are capped and polyadenylated: two spliced and one unspliced [14,15]. Full-length, unspliced 9.3 kb viral RNA is exported to the cytoplasm where it not only becomes the genome of progeny virions, but also acts as the mRNA template for Gag and Gag-Pol polyproteins [16]. This viral mRNA is presented to the host translation machinery with characteristics rarely observed among host cell mRNAs: a long 3' UTR, retained introns, and multiple open reading frames. As a result of these mRNA features, the full-length viral RNA should be recognized by the host NMD machinery and degraded; however, the RNA is stable with a half-life of ~7-20 hours [17,18].

Premature termination codons within the open reading frame of *gag *result in a decrease in unspliced viral RNA levels [19]. This decay relies upon the central NMD protein Upf1 and translation of the viral RNA, thereby implicating the NMD machinery in differentiating premature from natural termination codons in this unspliced viral RNA [20]. Thus, full-length viral RNA is not immune to host mRNA decay surveillance as has been observed for some intronless mRNAs in mammalian cells [21,22]. The *gag *open reading frame of RSV is removed from all spliced viral mRNAs; therefore a model that relies upon downstream exon junction complexes for recognition of a premature termination codon is unsatisfactory in the context of the RSV viral RNA. In fact, recent studies have suggested that an EJC is not required for recognition by NMD [22,23].

An alternative model in vertebrates proposes that NMD is induced when the termination codon is distant from the polyA tail and the polyA binding proteins [22-24]. The distance between the natural stop codon and the polyA tail is usually relatively short. In humans 80% of polyA tails are within 2 kb of the translation termination codon [25]. When a premature termination codon arises within the open reading frame, it would be a greater distance from the 3' polyA tail. In support of this model, some transcripts with long 3' UTRs are unstable and degraded by NMD [22,23,26-28]. The unspliced viral RNA is polycistronic, but Gag is the major protein product generated from this mRNA resulting in an apparent 3' UTR of over 7 kb. The average length of a 3' UTR in chicken cells is approximately 600 nucleotides, with over 80% of the polyA tails being within 1200 nucleotides of the translation termination codon [29,30]. Again, a model where the distance from a stop codon to the polyA tail would determine whether a termination codon is premature is difficult to reconcile in the context of RSV. Therefore, we propose that an alternative mechanism must exist to allow the NMD machinery to identify premature termination codons within RSV RNA.

During initial efforts to characterize the decay of unspliced RSV RNA, it was noted that deletions downstream of *gag *decreased unspliced viral RNA levels [31]. When 400 nucleotides downstream of *gag *are deleted or inverted, unspliced viral RNA levels are reduced to quantities comparable to viral constructs containing a premature termination codon within *gag *[18]. This *cis *RNA element was termed the Rous sarcoma virus stability element (RSE). Furthermore, when the RSE is inserted after a premature termination codon within the *gag *open reading frame, the viral RNA no longer undergoes decay [18]. This suggests that the RSE generates a signal to identify the correct termination codon.

We sought to define key RNA features of the RSE through directed mutagenesis of the virus. In this report we describe RNA sequence features that play a role in RSE function. These data suggest that the RSE is comprised of structure and sequence components with many redundant sub-elements. These elements function independently of the nucleotide sequence of the termination codon and the first nucleotide following the termination codon. Furthermore, the 3'UTRs of the other RSV open reading frames of the parental avian leukosis virus (ALV) may also function as stability elements.

## Results

### Truncations of the RSE reveal that the minimal functional element is 155 nts

Initial characterization of the RSE demonstrated that a 400 nt region of viral RNA downstream of the *gag *termination codon is important for maintaining stability of the full-length RSV RNA. Preliminary deletion analysis suggests that redundant or non-essential regions exist at the ends of the RSE since they can be deleted without significant effect on RSE function [18]. We carried out a directed approach to truncate the RSE and determine the 5' and 3' boundaries of the functional region. To facilitate cloning, we introduced unique restriction sites into the proviral vector sequences that flank the 400 nt RSE. The 5' site was placed eight nucleotides after the *gag *translation termination codon so that the immediate termination context of the stop codon would not be altered. This new proviral vector exhibited RNA levels comparable to other RSV wild-type viruses (data not shown).

**Figure 1:**
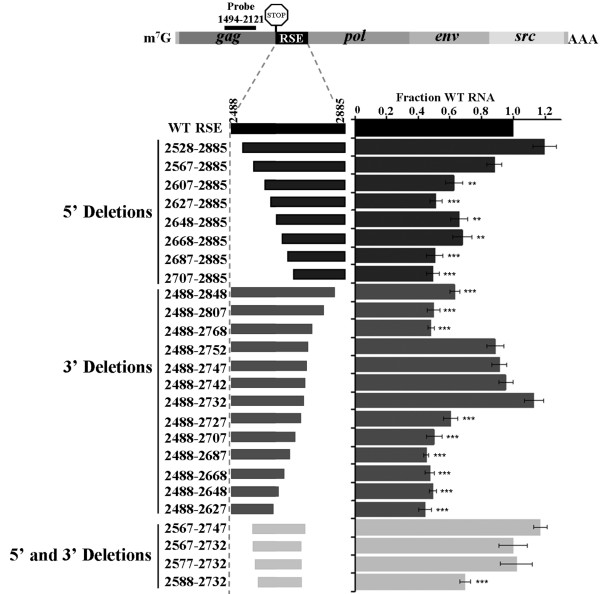
**5' and 3' truncations of the RSE indicated that the minimal functional element is 155 nts**. Truncations from the 5' and 3' end of the RSE were generated by PCR and cloned after the *gag *natural termination codon. Nomenclature of each construct indicates the nucleotide residue number. Diagram of deletions is to scale, with the location of the RPA probe used indicated. Each construct was transiently transfected into CEFs and RNA steady-state levels were assayed 48 hours later by RNase protection assay. Transfection efficiency was normalized using a wild-type viral loading control. RNA levels are reported as a fraction of wild-type RNA. Standard deviations are represented on the bar graph. Values represent the average of at least four experiments. Stars indicate a significant reduction compared to the wild-type virus (**: p < 0.001; ***: p < 0.0001).

Truncations to the 5' and 3' end of the 400 nt RSE were generated by PCR, and the amplicons were cloned into the wild-type virus after the translation termination codon. Steady-state RNA levels of these constructs were assayed by transient transfection of CEFs followed by an RNase protection assay using an RNA probe that is complementary to the *gag *coding region (Figure 1, diagram). The co-transfected loading control is a wild-type RSV construct that contains a deletion within the complementary region of the probe. As a result, the size of the protected probe band allows differentiation between the experimental and control viral constructs. After normalizing each experimental signal to its respective loading control, constructs that exhibit greater than 90% steady-state RNA levels when compared to wild-type RNA are considered stable. This analysis indicates that the ends of the functional element are at positions 2577 and 2732 of the viral RNA, a deletion of 75 nts from the 5' end of the RSE and 153 nts from the 3' end (Figure 1; 5' and 3').

The 5' truncations lie within the stem-loop of the highly structured pseudoknot (nts 2484-2584) that is required for transitioning the ribosome from the *gag *open reading frame to the *pol *open reading frame [11,32]. Since this pseudoknot could be deleted while the RSE retained function (constructs 2584-2885, 2567-2885 and [18]), we concluded that the pseudoknot structure does not play a role in RSE-mediated stabilization of the full-length viral RNA.

Initial truncations from the 3' end of the RSE were unstable (constructs 2488-2848, 2488-2807 and 2488-2768). We hypothesize that this is likely due to a disruption of the RSE RNA secondary structure in this region, including a previously described strong stem loop (nts 2755-2809; [33]). Furthermore, this element could be deleted while the RSE retained function (constructs 2488-2752, 2488-2747, 2488-2742 and 2488-2732). We conclude that although the sub-elements that are required for RSE function are flanked by two strong secondary structure elements in the wild-type virus, neither is essential for RSE function.

To ensure that redundant elements do not lie in the individually deleted regions, we deleted sequences from both the 5' and 3' ends of the RSE (Figure 1, Both). We found that the construct ranging from 2567 to 2732 was stable. In this minimal construct, a further truncation of 10 nucleotides from the 5' end to 2577 was still stable. Therefore, the RSE is functional as a minimal fragment of 155 nts that encompasses nts 2577 to 2732, henceforth called the minimal RSE.

**Figure 2:**
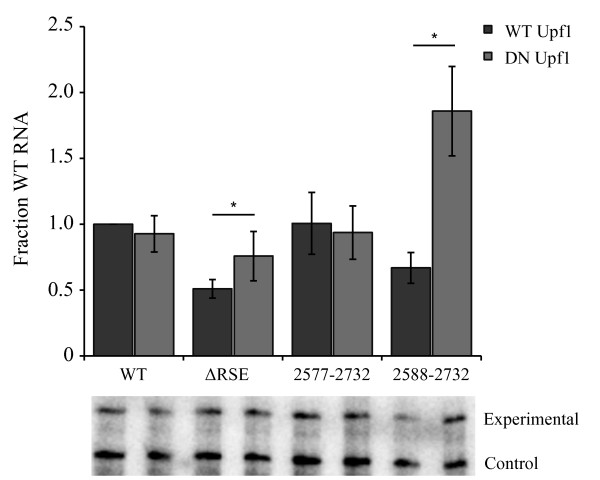
**The minimal RSE protected the *gag *stop codon from recognition by the NMD machinery**. Co-transfection of wildtype, ΔRSE, 2577-2732 (minimal RSE) and 2788-2732 with wildtype Upf1 or a dominant negative form of Upf1 (RR857GA). Each construct was transiently transfected into CEFs, and RNA steady-state levels were assayed 48 hours later by RNase protection assay. A representative RNase protection assay is shown, with the set of bands below each bar corresponding to the construct indicated directly above on the graph. The top band of the gel (experimental) is a fragment of *gag *probe protected during the RNase protection assay corresponding to the unspliced viral RNA from the experimental construct. The bottom band (control) is a wild-type viral loading control that protects a different sized fragment of the same *gag *probe due to a small deletion. Standard deviations are represented on the bar graph. Values represent the average of at least four experiments. A star indicates a significant reduction compared to the corresponding viral construct co-transfected with wild-type Upf1 (*: p < 0.01).

To confirm that the minimal RSE was still capable of insulating the *gag *termination codon from NMD recognition, we transiently co-transfected CEFs with either a wildtype or dominant negative form of Upf1 with each of the viral constructs (wildtype, ΔRSE, 2577-2732 and 2588-2732). As shown previously, the wildtype virus showed no significant change in the levels of unspliced RNA, while viral RNA lacking the RSE exhibited a 1.5 fold increase in the observed steady state RNA levels in the presence of mutant Upf1 (Figure 2). The minimal RSE (2577-2732) behaved like wild-type viral RNA. Furthermore, an RSE fragment slightly smaller than the minimal RSE (2588-2732) exhibited nearly a 3 fold increase in the level of unspliced RNA in the presence of mutant Upf1. This provides further support that the minimal RSE is the smallest functional unit because a smaller fragment appeared to be unable to protect the *gag *stop codon from recognition by NMD.

### Point mutations and deletions within the minimal RSE suggest multiple functional regions

To further characterize the sequence elements within the RSE we designed internal deletions and mutations based on the determined *in vitro *secondary structure of the 2660-2880 fragment [33]. The secondary structure of the minimal RSE, as determined by selective 2'-hydroxyl acylation analyzed by primer extension (SHAPE) (data not shown), was consistent with that of the larger RSE fragment [33]. We generated mutations that target the predicted single-stranded and stem-loop regions within the minimal RSE. A disruption of an essential RSE sub-element by these mutations would result in a loss of stability in the full-length viral RNA.

**Figure 3:**
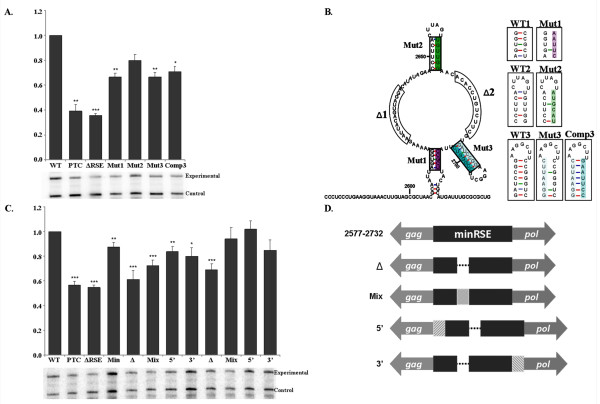
**Mutations within the minimal RSE suggest that multiple regions of sequence and structure contribute to function**. A. Steady-state RNA levels obtained by RNase protection assay of each point mutant. Point mutations within each of the predicted stem-loops resulted in a partial loss of stability relative to the wild-type viral RNA. A compensatory mutation (Comp3) of Mutant 3 that was predicted to reform the secondary structure did not recover wild-type levels. B. Structural diagram of the predicted secondary structure of the minimal RSE based on in vitro structure studies. The regions that were deleted (Δ1 and Δ2) and the stems that were mutated (Mut1-Mut3) are indicated. The boxes corresponding to each mutation indicate the sequence variation (highlighted region) while the structure displays the wild-type sequence. C. A 14 nucleotide (deletion 1) and a 12 nucleotide (deletion 2) single-stranded region of the minimal RSE were deleted and the level of viral RNA was assayed by RNase protection assay. Values are reported as a fraction of wild-type RNA. The deletions resulted in a partial loss of function. To determine whether spacing within the RNA was altered, sequence was added to the 5' or 3' end or at the deletion site. D. Diagram depicting the organization of the deletion constructs and the location of the sequence added back. **Δ**: The location of the deleted sequence is represented by a dotted line. **Mix**: The checked box represents deleted sequence that was scrambled and then added back at the deletion site. **5'**: The diagonally hatched box represents 10 nts of viral sequence added back to the 5' end of the minimal RSE sequence that contains the deletion. **3'**: The diagonally hatched box represents 10 nts of viral sequence added back to the 3' end of the minimal RSE sequence that contains the deletion. The added viral sequence is that which naturally lies just 5' or just 3' of the minimal RSE. The diagram is not to scale. Stars indicate a significant reduction compared to the wild-type virus (*: p < 0.01; **: p < 0.001; ***: p < 0.0001)

Individual point mutations were generated to disrupt the three predicted stem structures (Mut1, Mut2 and Mut3). The location of each mutation and the nucleotide changes are shown in Figure 3B. The mutations independently exhibited a partial loss of function, which resulted in an RNA steady-state level of 66.6 ± 0.03%, 80.0 ± 0.04% and 66.4 ± 0.04%, respectively, relative to wild-type (Figure 3A).

Previous studies indicate that stem 3 is readily formed under several *in vitro *experimental conditions and that it may be a key functional domain within the RSE [33]. To determine if the structure of this stem-loop is important, a compensatory mutation of Mutant 3 was generated that was predicted by the mFOLD software to restore the formation of the stem-loop structure. Reestablishing the stem-loop structure with a different sequence composition did not recover the loss of function observed for Mutant 3. The compensatory mutant exhibited a steady-state RNA level of 70.8 ± 0.04%; a value not significantly different from the single mutant (Mut 3, 66.4 ± 0.03%) (Figure 3A). This suggests that if the determined stem-loop structure is important for function; the sequence composition of the stem is as well.

To assess the importance of the proposed single-stranded regions, we generated a 14 nucleotide deletion (Δ1) and a 12 nucleotide deletion (Δ2) (Figure 3B). Both deletions resulted in a reduction in the amount of full-length viral RNA to 61.3% and 69.1%, respectively (Figure 3C). In this experiment, these values were comparable to that observed for the viral RNA bearing a PTC or one lacking the RSE. To ensure that the internal deletions do not alter the spacing of individual RNA sub-elements within the RSE or RNA features flanking the RSE, we added back scrambled sequence at the deletion site (Figure 3D, Mix). This resulted in recovery of wild-type RNA levels for Δ2 and a partial recovery for Δ1. These deletions indicate that the spacing between RNA elements is altered or that a minimal size of 155 nts is required for RSE function.

As a means of understanding whether the spacing to an element upstream or downstream of the minimal RSE causes the reduction in RNA levels observed from the deletion constructs, 10 nts of viral sequence were added back to either the 5' or 3' end of the minimal RSE with the deletion (see diagram in Figure 3D). Addition of sequence to the 5' end, and to a lesser extent to the 3' end, recovered wild-type RNA levels (Figure 3C). The same pattern was observed for both deletions, but the recovery for Δ1 remained slightly below wild-type levels. It is possible that the spacing of an RSE sub-element 3' to the deletion site is altered relative to an RNA feature 5' of the RSE. The incomplete recovery for Δ1 was likely due to the different size of the deletions.

In summary, these data suggest that the minimal RSE is a complex element with many sub-elements contributing to the function of the RSE to maintain a required spacing and facilitate formation of the RNA secondary structure. These different sub-elements seem to be dependent upon each other such that changes to any of these features result in a partial loss of RSE function.

### A termination codon within the RSE promotes decay of the viral RNA only when the *gag *stop codon is readthrough

Deletion of sequences within the minimal RSE suggests that the spacing of sub-elements within and flanking the RSE are important for maintaining function. This suggests that truncation of the RSE, as was done in Figure 1, may be limited in its utility in determining the 5' functional boundary of the RSE. One cannot differentiate whether a shorter truncation is due to a critical reduction in the spacing of the functional RSE to an upstream RNA feature or removal of a sequence implicitly essential to RSE function. As an alternative approach to determining the 5' boundary of the RSE, we inserted stop codons into the RSE and forced readthrough of the *gag *termination codon by inserting a single nucleotide to shift the ribosome into the *pol *reading frame. As shown previously, premature termination codons within the *pol *reading frame at nucleotide positions after 3004 will undergo decay, but only when the ribosome does not stop at the *gag *termination codon [18]. We hypothesize that if the stop codon is upsteam of a functional RSE, then it will not be recognized by the NMD machinery; and as a result, the RNA would be stable.

**Figure 4:**
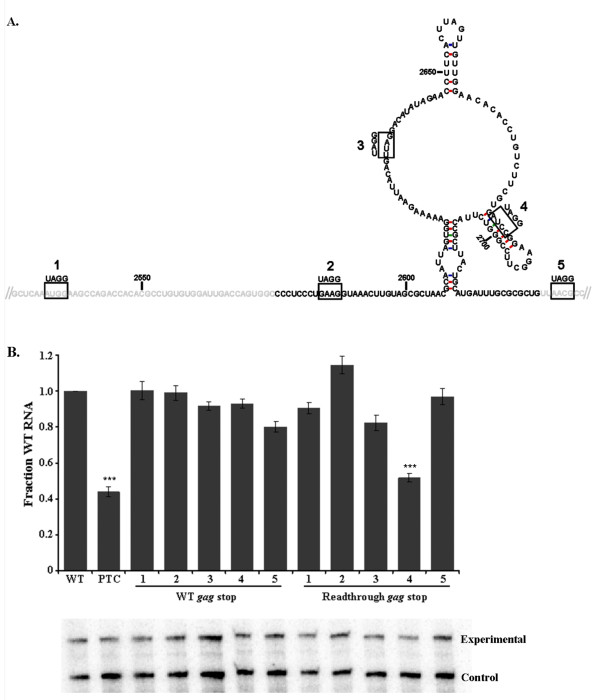
**A premature termination codon inserted within the RSE at position 2685 underwent decay when readthrough of the gag stop codon was forced**. A. Schematic of stop codon locations within the RSE. Premature termination codons were generated within the RSE at positions 2535, 2586, 2631, 2685 and 2736, named 1-5, respectively. The black font represents the minimal RSE as determined by truncations. Text in grey is RSE sequence flanking the minimal RSE. B. The RSE containing these mutations was cloned into two constructs, one with the natural *gag *termination codon sequence (WT *gag *stop) and the other with a single nucleotide insertion preceding the *gag *termination codon that forced readthrough into the *pol *open reading frame (Readthrough *gag *stop). Only PTC4 at position 2685 underwent decay in the readthrough construct. A representative RNase protection assay is shown, with the set of bands below each bar corresponding to the construct indicated directly above on the graph. Stars indicate a significant reduction compared to the wild-type virus (***: p < 0.0001)

Five stop codons were inserted into the RSE at nucleotide positions 2535, 2586, 2631, 2685 and 2736; numbered 1-5, respectively (Figure 4A). The unspliced viral RNA generated from each construct was stable when translation termination occurred at the *gag *stop codon, indicating that RSE function was not disrupted by any of the single point mutations (Figure 4B, WT *gag *stop). When a single nucleotide insertion immediately 5' of the stop codon constitutively forced the ribosome past the *gag *stop codon and into the *pol *open reading frame, the termination codon at position 2685 resulted in a reduction in the steady state levels of unspliced viral RNA (Figure 4B, Readthrough *gag *stop 4). The 5' boundary of the functional RSE as determined by truncations is 2577; however, a termination codon at position 2631 was still protected from NMD recognition (Figure 4B, Readthrough *gag *stop 3). This suggests that the sequence between 2577 and 2631 was likely required to maintain a particular spacing in the context of the minimal RSE and can act to enhance the ability of the RSE to protect the stop codon from recognition by NMD.

Additionally, we observed that a stop codon at nucleotide position 2736 (Figure 4B, Readthrough *gag *stop 5), a mere four nucleotides after the 3' boundary of the minimal RSE, did not undergo decay. This suggests that the RSE may be able to function not only downstream of a termination codon, but also when located upstream. Alternatively, these data may highlight the presence of redundant sequence elements downstream of the minimal RSE sequence that are present within the context of the full 400 nt RSE element. This property is distance dependent because termination codons at nucleotide positions 3004, 3739 and 4618 were previously shown to be recognized by NMD and that the resulting viral RNA is unstable [18].

These data suggest that the region containing the key sub-elements of the RSE lie within 100 nts (2631-2732). The 100 nucleotide core fragment encompasses the structural features of the minimal RSE that we have herein named stem 2, single-stranded region 2 and stem 3; although, sequence flanking this region may enhance RSE function when present in the full-length viral RNA. This provides further evidence that the minimal RSE (2577-2732) is the functional region that is facilitating the RSV viral RNA stabilization and NMD insulating phenotype that we have previously described [18]. Furthermore, the RSE may be able to function independently of its position relative to the stop codon, since it appears to function when placed upstream of a stop codon.

### Neither the sequence of the stop codon nor the fourth nucleotide affects RSE function

**Figure 5:**
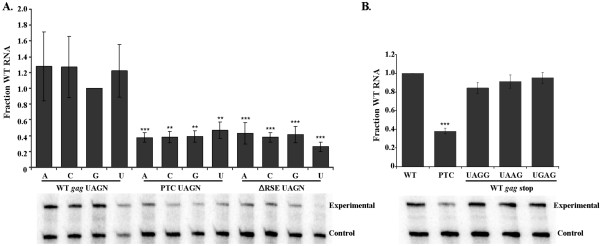
**The immediate termination codon context did not affect levels of the unspliced viral RNA**. A. The fourth nucleotide of the termination codon signal was altered to each of the four possible ribonucleotides. This was done at the *gag *stop codon, with and without the RSE and at a premature termination codon at nucleotide 1250. Steady-state levels of RNA were assayed by RNase protection assay. These mutations did not significantly affect the stability of the wild-type viral RNA (WT *gag *UAG), nor the efficiency of decay of the RNAs bearing a premature termination codon within *gag *(PTC UAG) or lacking the RSE (ΔRSE UAG). B. The stop codon of the *gag *termination codon (UAG) was altered to each of the other two stop codons (UAA and UGA). The level of steady-state viral RNA was not affected, as assayed by RNase protection assay. Representative RNase protections are shown. Stars indicate a significant reduction compared to the wild-type virus (**: p < 0.001; ***: p < 0.0001)

Work from the Jacobson lab suggests that one of the termination signals that promotes NMD recognition of a stop codon in yeast is inefficient translation termination [34]. A key feature in determining efficiency of translation termination is the immediate stop codon context [35,36]. The stop codon context is comprised of the stop codon itself (UAA, UAG or UGA) and the nucleotides following the stop codon and most importantly, the first nucleotide following the stop codon [37,38]. To test if the immediate stop codon context has an effect on the level of viral RNA decay observed, we altered the first nucleotide after the UAG stop codon at a premature stop codon within *gag*, and after the natural *gag *stop codon, with and without the RSE present downstream. In none of these cases was the amount of RNA observed altered (Figure 5A). This effect was also independent of the stop codon used, as viral constructs that have the UAG *gag *stop codon altered to either UAA or UGA exhibited no difference in viral RNA levels (Figure 5B). We conclude that the sequence of the stop codon has no effect on RSE function. This suggests that the RSE dependent determination of premature termination occurs after stop codon recognition.

### Potential stability elements exist downstream of the other viral UTRs

**Figure 6:**
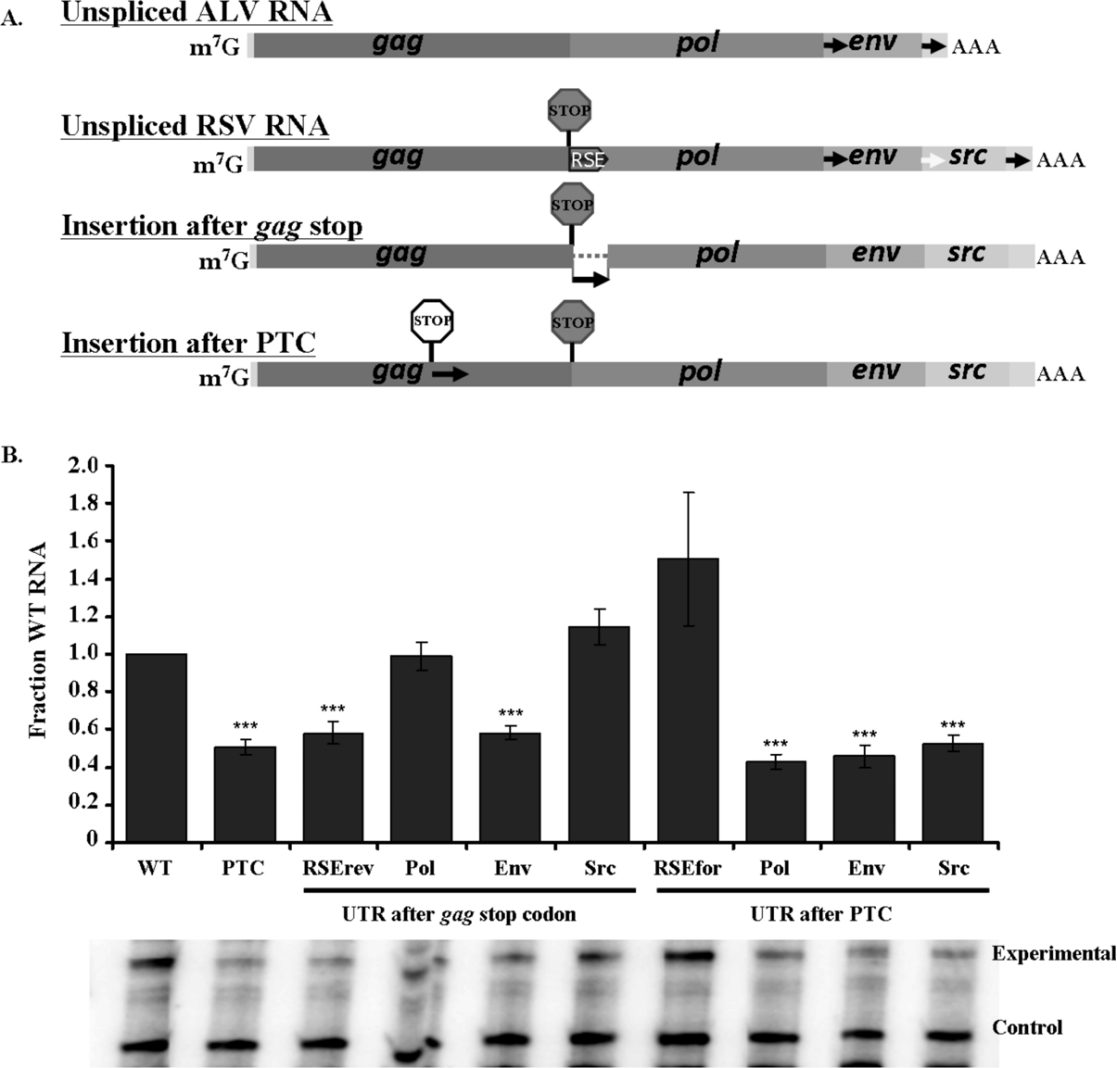
**The natural viral UTRs can substitute for the RSE after the *gag *termination codon, but not after a premature termination codon**. A. Diagram of the viral UTR cloning strategy. The UTRs of *pol*, *env*, and *src *(black arrows) were cloned after the *gag *natural stop codon (grey octagon) and after a premature termination codon (white octagon) within *gag *at position 1250. B. The *pol *and *src *UTRs were able to maintain wild-type RNA levels when placed after the *gag *natural termination codon. None of the UTRs other than the RSE was capable of stabilizing the RNA when placed after a premature termination codon. RNA levels were assayed by RNase protection assay. Representative gels are shown below each graph. Stars indicate a significant reduction compared to the wild-type virus (**: p < 0.001; ***: p < 0.0001)

In addition to *gag*, RSV contains three other open reading frames; *pol, env *and *src *[14]. While Env and Src are expressed from two separate spliced transcripts, Pol is generated by a programmed -1 frameshift that repositions the ribosome out of the *gag *reading frame and into the *pol *reading frame [16]. This rare translation event occurs only about 5% of the time, meaning that Gag is the predominant protein product. To determine if the other RSV genes have stability elements downstream of their respective stop codons, we cloned 400 nts from the beginning of the 3' UTRs after the *gag *stop codon in lieu of the RSE, as well as after a premature termination codon in *gag *(Figure 6A). We found that the 3' UTRs of *pol *and *src *were able to substitute for the RSE after the *gag *termination codon, while the negative control antisense RSE and the *env *3' UTR could not (Figure 6B).

In comparison to other simple retroviruses, such as ALV shown in Figure 6A, RSV has an additional open reading frame located at its 3' end. Unique to RSV, the 3' UTR of *env *is actually the coding region of the cellularly-derived *src *gene. Src is a cellular proto-oncogene that was incorporated into the genome of the parent virus ALV [39]. We hypothesize that these stability elements are located mainly in 3'UTRs and not in coding regions. Furthermore, in order for a viral RNA element to co-evolve to interact with cellular machinery, we would expect only native viral sequences to be capable of being a stabilizing element. Since the 3'UTR of RSE *env *is a newly acquired cellular coding region, it is not expect to possess the ability to stabilize the unspliced RSE RNA.

Surprisingly, none of the viral UTRs other than full length *gag *RSE was capable of stabilizing the RNA when placed after the premature termination codon in *gag *(Figure 6B). The same effect was observed whether the RSE was present downstream of the *gag *natural termination codon or not (data not shown). This may be indicative of several possible scenarios. First, the RSE itself may be more efficient at identifying a translation termination codon in a heterologous context such as at a premature termination codon. When the other viral UTRs are present, additional sequences upstream of the natural *gag *stop codon, which are absent from a premature stop codon, may contribute to prevention of NMD recognition. Secondly, the 3'UTRs of the other viral termination codons may not function by the same mechanism as the RSE.

The RSE may be more robust in our assay than the other viral 3' UTRs because Gag is the predominant viral protein product, and it has been selected to be more efficient at preventing recognition of the *gag *termination codon by NMD. At least 20 fold less Pol, Env and Src protein products are produced relative to Gag; therefore an efficient signal at the other stop codons may not be absolutely required [40]. Furthermore, the 3'UTRs of Env and Src are approximately 2 kb and 0.6 kb upstream of the polyA signal, which may be close enough to the polyA tail and polyA binding protein to allow the termination codons to be partially protected from NMD.

### The minimal RSE functions only after the natural *gag *stop codon

**Figure 7:**
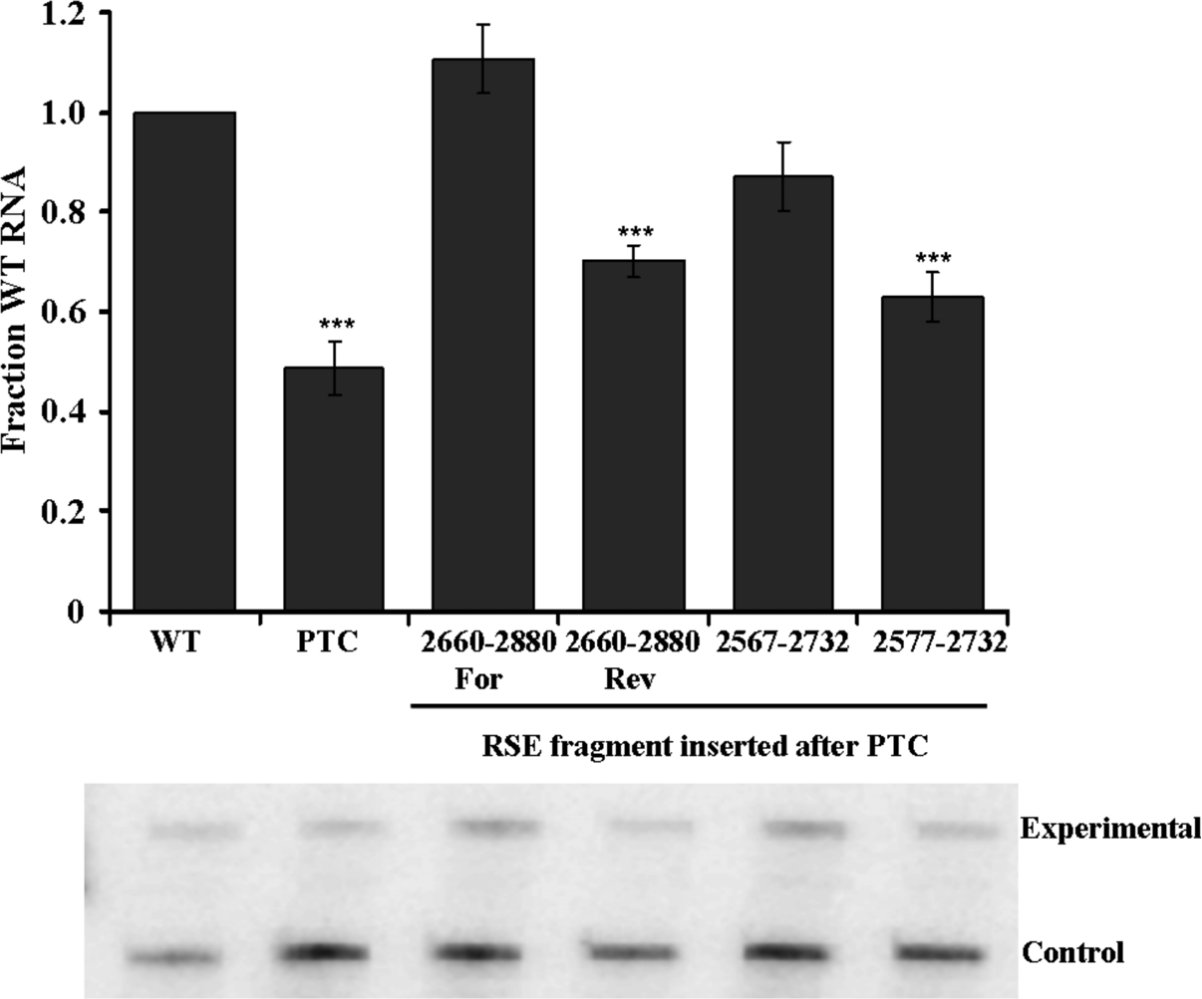
**The minimal RSE functions only after the *gag *termination codon**. The RSE was cloned in the forward (2660-2880 For) and reverse orientation (2660-2880 Rev) after a premature termination codon within *gag *at position 1250. The minimal RSE (2577-2732) and a slightly longer RSE fragment (2567-2732) were cloned after the same premature termination codon. 2660-2880 For is a previously described functional fragment of the RSE (Cfor; [25]). The minimal RSE is unable to stabilize the RNA when placed after a premature termination codon within *gag*. RNA levels were assayed by RNase protection assay. Representative gels are shown below each graph. Stars indicate a significant reduction compared to the wild-type virus (***: p < 0.0001)

The data from the other viral UTRs suggest that there may be enhancing elements either flanking the primary functional region of the RSE or 5' of the *gag *termination codon. We hypothesize that the minimal RSE is a rudimentary version of the fully functional RSE in which redundant and enhancing sequences have been removed. Therefore, if the minimal RSE is moved from its natural context, it may no longer to be able to function. In accordance with this model, the minimal RSE was unable to act like the wild-type RSE at a premature stop codon within *gag *(Figure 7A). Steady state RNA levels were reduced to levels comparable to the premature termination codon alone. Furthermore, when as little as 10 nucleotides of additional RSE sequence were added to the 5' end of the minimal RSE (2577-2732), a modest but reproducible increase in the level of RNA was observed. This suggests that the structure of the RSE may be influenced by the surrounding sequence context. This enhancement was absent when the same truncated RSE fragments were tested after the natural *gag *termination codon (Figure 1, compare 2577-2732 and 2567-2732). This is consistent with the ability of flanking sequences to enhance the formation of the functional structure of the minimal RSE at the natural *gag *termination codon.

## Discussion

### The RSE and sequences upstream of the *gag *stop codon contribute to correct stop codon identification

Within the minimal RSE element, point mutations and deletions were used to characterize sequences and secondary structure elements. All of the mutations tested resulted in a partial reduction in RSE function, which suggests that the sequence and structure of multiple sub-elements within the RSE may work together to generate a signal or recruit a protein that identifies the correct stop codon.

An alternative interpretation of the deletion and truncation data is that the RSE is merely a nucleotide spacer of a defined size, in this case approximately 155 nts. Additional deletions that reduce the size of the RSE below this critical limit would be unstable because the *gag *termination codon would be moved closer to a yet uncharacterized destabilizing element further downstream from the RSE. However, evidence from our lab demonstrates that the RSE can function as a genuine stabilizing element. First, as premature termination codons inserted into the gag open reading frame approach the natural stop codon, the amount of decay observed decreases [31]. This suggests that there is a signal identifying the natural termination codon. Furthermore, the RSE can be moved downstream of a premature termination codon within *gag *to stabilize the RNA [18]. Thirdly, if the RSE were a spacer for a downstream element, a sequence of any composition should work. In this study we show at least 2 sequences (the *env *3' UTR and the reverse sequence of the RSE) were unable to substitute for the RSE. Therefore, although we cannot exclude the possibility that there is a destabilizing element downstream of the RSE, this RNA sequence exhibits the ability to identify the correct termination codon.

### The other viral open reading frames may also have stability elements

The deletions within the minimal RSE suggest that there may be sequences upstream of the *gag *stop codon that contribute to RSE function. This is supported by the data from the other viral UTRs at the premature stop codon. The viral UTRs *pol *and *src *were able to substitute for the RSE at the natural termination codon where their ability to prevent NMD recognition was enhanced by flanking sequences. However, when the viral UTRs were placed after a premature termination codon, this enhancement was absent and they were no longer able to substitute for the RSE.

The 3' UTR of *env *was not able to substitute for the RSE after the *gag *termination codon. Several previous studies indicate that regulation of mRNA stability encoding the *env *gene product may be unique. First, a study by Simpson and Stoltzfus [41] showed that the *src *mRNA, but not the *env *mRNA, undergoes decay when premature termination codons are generated by deletions that cause frameshifts. Second, according to Stoltzfus *et al*. [17], the full-length viral RNA decays with a half-life of 7.5 hours, while the spliced *env *message is more stable with a half-life of 10 hours. They propose that the membrane association of polysomes containing *env *mRNA may stabilize it relative to the viral mRNAs which are on free cytoplasmic polysomes [17,42]. This increased protection at the membrane may shield the *env *viral mRNA from NMD detection thereby obviating the need for an NMD insulator sequence similar to the RSE.

### The Rous sarcoma virus as a tool to study nonsense-mediated mRNA decay

Retroviruses have long been a useful tool for studying cellular and molecular biology *in vivo*. Their need to hijack host cell processes in order to replicate and produce progeny provides scientists with a valuable tool with which to better understand all areas of nucleic acid production and trafficking. Elements within retroviral RNA modulate RNA splicing efficiency, RNA export from the nucleus, translation, mRNA stability and assembly of virions [43,44]. Thus, multiple layers of control are used by retroviruses at the level of RNA which serve as a compact resource for interaction with host proteins and pathways in the nucleus and cytoplasm. RSV provides a unique perspective with which to understand NMD.

Recently, numerous cases have been reported in the literature in which the exon junction complex is not absolutely required for identification of a premature stop codon by NMD, but rather it may simply act as an enhancer, with other mRNA features, such as the polyA tail, providing the underlying signal [22-24]. Although the evidence is compelling, most of these studies rely on artificial constructs to study NMD in the absence of splicing or to alter the distance from the polyA to the stop codon. A retrovirus such as RSV has evolved to possess all of these features naturally; therefore it can act as an elegant reporter for the mechanism of NMD recognition of premature stop codons on an unspliced RNA. Furthermore, a better understanding of retroviral RNA elements can enhance the efficacy and potency of retroviral vectors used in medicine where open reading frames are deleted or altered without a true depth of understanding of the underlying regulatory RNA sequences.

### The RSE identifies the correct translation termination codon

The RNA stability element within the Rous sarcoma virus prevents NMD recognition and decay of the full length viral RNA, despite several characteristics uncommon in cellular messages. From the data obtained from this study, we can begin to establish some basic features essential to the mechanism by which the RSE may facilitate NMD evasion.

Using artificial constructs, it was shown that a fold-back mechanism can prevent NMD recognition of a termination codon [23]. This model would suggest that RSE RNA base-pairing with sequences proximal to the 3' end would bring the polyA tail and associated factors in proximity to the translation termination codon. It seems unlikely that the viral NMD evasion is due to a fold-back mechanism since multiple insertions and deletions as small as 10 nts are capable of significantly reducing RSE function.

### Preliminary model for RSE function

**Figure 8:**
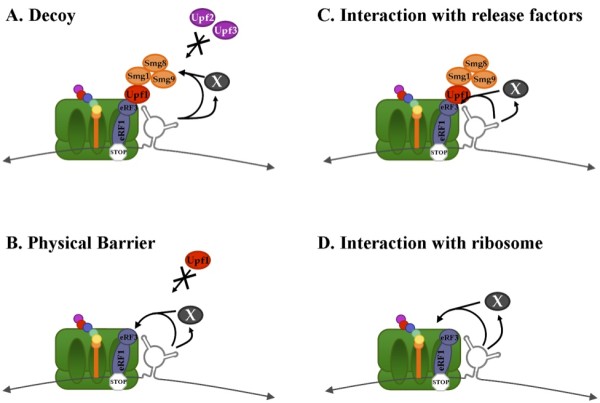
**The RSE may act as an NMD insulator**. Either through a direct interaction with the RNA, or mediated through a protein, the RSE may act as an NMD insulator. A. The RSE may act as a decoy to conceal the *gag *stop codon from association with some NMD factors or to interact with Smg8/9 to keep Smg1 inactive. B. The RSE may interact with the translation termination machinery at a site that overlaps with that of Upf1, such that the NMD machinery can no longer associate with eRF3. C. The RSE may interact with eRF3 to promote translation termination. D. The RSE may interact directly with one of the ribosomal subunits to induce a conformational change that favors translation termination.

The dependence upon the sequence for function of the RSE suggests that the RNA may be interacting with a protein. In this model the RSE is a recognition site for an NMD insulator complex. This protein complex may create a boundary which prevents communication between the translation competent ribosome and the NMD machinery (Figure 8). We can envision this complex functioning in two ways. First it may act as a decoy, which interacts with the NMD machinery such that it is no longer able to associate with the release factors (Figure 8A). Alternatively it may act as a physical barrier by interacting with eRF3 at a site that overlaps with that of the Upf1 recognition site, thereby preventing productive NMD complex formation (Figure 8B). Interestingly, this interaction between the RSV viral RNA and cellular proteins may represent another example of the virus hijacking a cellular mechanism. Long 3' UTRs exist in natural mRNAs which evade NMD such as Cript1 and Tram1 [22]. These mRNAs may associate with the same factors as the RSE.

However, if the RSE is able to function upstream of a termination codon, it may not be possible for a protein to associate with the RSE since a ribosome would remove the protein from the RNA during translation. In order for the RSE to function upstream and downstream of the termination codon, the RNA itself may fold into a tertiary structure and interact directly with the termination competent ribosome arrested at the stop codon to prevent association with the NMD machinery.

We have also described in this study a size dependence of the RSE, such that shortening the RSE below 150 nucleotides results in a loss of function. Although from the deletion data we propose that it is likely that additional elements lie upstream, it is also possible that a particular size of the RNA is required in 3D space that allows for interaction with the distal protein factors. Presumably if the RNA is interacting with the translation termination machinery and Upf1, either directly or through a yet unidentified protein, this interaction would need to span the distance from the base of the RSE RNA to the top of the A site where the release factors reside (Figure 8C).

Alternatively, an interaction with a ribosomal subunit distal to the A site may facilitate a conformational change in the ribosome that favors translation termination in the presence of the eukaryotic release factors (Figure 8D) [45-47]. If the RSE possesses the ability to function downstream and upstream of a termination codon (Figure 4), this is the most likely model because the RSE may be able to contact the ribosome regardless of its location and would not require an additional protein factor.

## Conclusions

This paper describes a minimal 155-nt RNA sequence downstream of the RSV *gag *termination codon that makes the full-length RSE viral RNA immune to NMD. Additionally, we have demonstrated that RSV has RNA stability elements immediately downstream of the open reading frames of *gag*, *pol*, and *src*. We propose that these viral stability elements act as insulators, masking the authentic termination codons from the NMD machinery. Furthermore, this study provides more evidence that the exon junction complex is not required for identification of a premature termination codon. This novel type of RNA regulatory structure will likely also be found in some cellular mRNAs. Future studies will focus on the role of protein factors in RSE function, namely assessing the impact of the other NMD factors on decay of the unspliced viral RNA.

## Materials and methods

### Cell culture and transfections

Secondary chicken embryo fibroblast (CEF) cultures were grown at 39°C and 5% CO_2 _in medium 199 supplemented with 2% tryptose phosphate broth, 1% chick serum, 1% calf serum and 1% penicillin-streptomycin. Transient transfection assays were performed with DEAE dextran at a concentration of 200 μg/mL in serum free medium 199 as previously described [48]. Cells were transfected in 6 cm dishes with 3 μg of DNA when they were 90% confluent. Total cell RNA was harvested from CEFs using RNA-Bee as per the manufacturer's instructions. The Upf1 constructs (hUpf1 and RR857GA) were a generous gift from Hal Dietz and are described previously [49].

### RNase protection assay

*In vitro *transcription of the *gag *probe was performed from a T7 DNA template and radiolabeled with [a-^32^P]GTP using viral sequences previously described [20]. Whole cell RNA (10 μg) was resuspended in 30 μL of 80% formamide hybridization solution (80% [vol/vol] deionized formamide, 40 mM piperazine-N, N'-bis(2-ethanesulfonic acid) [pH 6.7], 1 mM EDTA, 0.4 mM NaCl) and ~250 000 cpm of *gag *probe was added. RNAs were denatured at 95°C and incubated at 42°C for 16 hrs. 300 μL of RNase digestion buffer (10 mM Tris-HCl [pH 7.5], 300 mM NaCl, 5 mM EDTA, 10 U of RNase T1/mL and 5 ug of RNase A/mL) was added and then incubated at 33°C for 45 min. Sodium dodecyl sulfate and proteinase K were added to final concentrations of 0.6% (vol/vol) and 0.14 mg/mL, respectively, followed by a 20 min incubation at 37°C to stop the RNase digestion. The samples were extracted with an equal volume of phenol-chloroform-isoamyl alcohol (25:24:1) followed by ethanol precipitation. RNAs were resuspended in 95% formamide loading dye (95% [vol/vol] deionized formamide, 0.02% bromophenol blue, 0.02% xylene cyanol) and denatured for 3 min at 95°C. Samples were electrophoresed on a 6% acrylamide-8 M urea sequencing gel. RNA levels were quantified using a Phosphoimager and Imagequant (GE).

### Viral constructs and cloning

All RSV nucleotides correspond to the following NCBI entry [Genbank: NC_001407]. The 10.8 viral plasmid used to generate each of the constructs contains a deletion in the nucleocapsid region of the *gag *gene [50]. The construct PTC-RSEfor has been described previously [18]. To generate unique restriction sites EagI and SpeI that flank the RSE, two sequential quick-change reactions were performed with the following primers.

Eag1 QCF 5' CTTGACAAATTTATAGGGAGGGCGGCCGTTCTCACTGTTGCGCTAC

Eag1 QCR 5'GTAGCGCAACAGTGAGAACGGCCGCCCTCCCTATAAATTTGTCAAGC Spe1 QCF 5' CGCGAAGCTTTTGCATTTACACTAGTCTCTGTGAATAACCAGGCCC

Spe1 QCR 5' GGGCCTGGTTATTCACAGAGACTAGTGTAAATGCAAAAGCTTCGCG

This new wild-type vector was called E/S. To generate each of the truncations or viral UTR insertions after the *gag *stop codon, PCR primers were designed that possessed an EagI recognition site in the forward primer and an SpeI recognition site in the reverse primer. Amplicons and the E/S wild-type viral vector were digested with EagI and SpeI. The vector was treated with calf intestinal phosphatase. Digested vectors and amplicons were purified with the Qiagen gel extraction kit from a 1.5% agarose gel. These were used in ligation reactions and transformed into E. coli. Positive clones were screened by digestion and confirmed by sequencing. Each mutant was then selected and grown for plasmid purification.

Sequences cloned after premature termination codons were inserted into a unique AatII site at nucleotide 1250 of 10.8. These sequences were amplified from the 10.8 vector with flanking AatII sites and a UAG stop codon at the 5' end in frame with the *gag *gene.

To generate the stop codon changes at the PTC and the natural stop codon, primers were designed to contain the mutations. A region between a unique AatII recognition site at 1250 and the unique EagI site at 2488 was amplified. This PCR fragment was then digested and cloned into the corresponding sites in the E/S vector. Positive clones were screened by digestion and sequencing. The following primers were used. Changes from the wild-type sequence are in bold. Where an N is indicated, a separate primer was generated with each of the four possible deoxynucleotide residues at that position.

#### Wild-type stop codon

AatII WT for CGCATGACGTCACGAATCTAATGAGAG

EagI UAAN rev CGAACGGCCGCCCTC**N**TTATAAATTTGTCAAGCGG

EagI UGAN rev CGAACGGCCGCCCTC**N**TCATAAATTTGTCAAGCGG

EagI UAGN rev CGAACGGCCGCCCTC**N**CTATAAATTTGTCAAGCGG

#### *Gag *stop codon with ΔRSE

AatII WT for CGCATGACGTCACGAATCTAATGAGAG

SpeI UAGN rev CGAAACTAGTCCCTC**N**CTATAAATTTGTCAAGCGG

#### PTC at 1250

AatII UAGN for CGCATGACGTCTAG**N**ATCTAATGAGAG

EagI UAGG rev CGAACGGCCGCCCTCGCTATAAATTTGTCAAGCGG

Premature termination codons were introduced into the RSE by quickchange mutagenesis of the E/S vector with the following primers. Briefly, the wild-type E/S vector was amplified by PFU Turbo (Stratagene). Forty units of Dpn1 was added directly to the PCR reaction and incubated for 30 min at 37°C. 4 μL of this solution was transformed into E. *coli*. Positive clones were screened by digestion and sequencing. Only the forward primers are shown. The PTC is shown in square brackets, with changes to the wild-type sequence in bold.

QCPTC2535 CATCTGGCTATTCCGCTC[**T**A**GG**]GGAAGCCAGACCACAC

QCPTC2586 GTGGCCCCTCCCT[**T**A**G**G]GTAAACTTGTAGCGCTAACGC

QCPTC2631 CGCAATTAGTGGAAAAAGAATTA[**T**AG**G**]TAGGACATATAGAACCTTCACTTAGTTGTTGG

QCPTC2685 GAACACACCTGTCTTCGTG[**TAGG**]GGAAGGCTTCCGGG

QCPTC2736 CATGATTTGCGCGCTGTT[**T**A**G**G]CCAAGCTTGTTCCTTTTGG

## Abbreviations

RSV: Rous sarcoma virus; UTR: untranslated region; CEF: chick embryo fibroblast

## Competing interests

The authors declare that they have no competing interests.

## Authors' contributions

JBW designed and performed experiments, analyzed and interpreted data, and drafted the manuscript. KLB contributed to data interpretation and reviewed and edited the manuscript. All authors read and approved the final manuscript.
